# Trends in breast cancer incidence among women under the age of forty

**DOI:** 10.1038/sj.bjc.6603919

**Published:** 2007-08-07

**Authors:** F Levi, V-C Te, M Maspoli, L Randimbison, J-L Bulliard, C La Vecchia

**Affiliations:** 1 Cancer Epidemiology Unit and Cancer Registry of Vaud, Institut de médecine sociale et préventive (IUMSP), Centre Hospitalier Universitaire Vaudois et Université de Lausanne, CHUV-Falaises 1, Lausanne 1011, Switzerland; 2 Cancer Registry of Neuchâtel, Av. des Cadolles 7, Neuchâtel 2000, Switzerland; 3 Laboratory of Epidemiology, Istituto di Ricerche Farmacologiche ‘Mario Negri’, Via La Masa 19, Milano 20156, Italy; 4 Istituto di Statistica Medica e Biometria ‘GA Maccacaro’, Università degli Studi di Milano, Via Venezian 1, Milano 20133, Italy

**Sir,**

Bouchardy *et al* (2007) reported that the Geneva Cancer Registry records show a substantial rise in breast cancer incidence in women under the age of 40 years in the period 2002–2004, and called for confirmation of their finding in other population-based cancer registries. We therefore abstracted data from the cancer registries of two other French-speaking Swiss Cantons, Vaud and Neuchâtel (Census populations (December 2000) of about 620 300 and 165 700, respectively; Levi *et al*, 2003a, 2003b).

Table 1 gives annual number of registered breast cancer cases in women aged 25–39 years and the corresponding age-standardised rates (on the European standard population) from the two registries over the period 1995–2005. Over the last 3 years, rates were around 33/100 000, and there was some rise over the 11-year calendar period considered (the per cent annual rise was 2.7 in Vaud, 1.6 in Neuchâtel, both not statistically significant), but there was no substantial nor sudden increase in most recent years.

When older age groups were examined, some upward trends in breast cancer registrations were observed, particularly in young and middle-aged women in Vaud, but there was again no indication of a recent sudden rise. At ages 40–49 years, rates were 118.5 in 1995 and 184.9 in 2005, the rates were 307 and 338.2 at ages 50–69 years, 339.9 and 338.4 at ages 70–79 years, and 358.2 and 366.1 at ages 80 and above. Corresponding rates for Neuchâtel were 126.5 in 1995 and 186.4 in 2005 at age 40–49 years, 392.8 and 319.7 at age 50–69 years, 251.0 and 423.7 at age 70–79 years, and 374.4 and 422.5 at ages 80 years and above.

These data from the Vaud and Neuchâtel Cancer Registries confirm therefore that breast cancer incidence registration has been rising over the last decade, particularly in young and middle-aged women. This rise is largely or totally due to increased detection. In fact, an organised mammography screening programme has been in operation in Vaud since 1999 for women aged 50–70 years (Bulliard *et al*, 2003), and the use of mammography has increased also in younger women. Breast cancer mortality has fallen by 30% in French-speaking Swiss women between 1990 and 2002 (Bulliard *et al*, 2006), further indicating that any appreciable real rise in incidence is unlikely. Breast cancer incidence below age 40 has been stable in the USA between 1999 and 2003 (Morbidity and Mortality Weekly Report, 2007).

The present data do not confirm the existence of a substantial rise in breast cancer incidence in younger women in recent years. The upward trends in Geneva may therefore be due to the play of chance, since they were based only on 50 cases registered in 2003-2004, or the detection of prevalent cases due to increased use of mammography in young women in recent years.

## Figures and Tables

**Table 1:** Trends in numbers and age-adjusted (European standard population) incidence rates of breast cancer per 100 000 women aged 25–39 years at diagnosis in the Cantons of Vaud and Neuchâtel, and annual percent change over the period 1995–2005

	**Year**											
**Cancer registry**	**1995**	**1996**	**1997**	**1998**	**1999**	**2000**	**2001**	**2002**	**2003**	**2004**	**2005**	**Annual % change^a^** **(95% CI)^b^**
*Vaud*												
Number	16	15	23	27	26	30	23	21	28	22	26	+2.7
Rate/10^5^	22.4	20.8	31.4	36.5	34.7	40.1	30.2	27.4	36.2	27.9	34.1	(−0.94–6.4)
												
*Neuchatel*												
Number	5	6	6	4	6	5	4	5	6	8	6	+1.6
Rate/10^5^	26.5	30.8	31.0	20.7	30.7	25.4	20.0	24.7	29.7	41.4	31.0	(−2.3–5.5)

a Computed over the period 1995–2005 using a standard log-linear model.

b CI=confidence interval.
